# “Double trouble”: the impact of iron infusion and antiresorptive therapy on calcium-phosphate homeostasis

**DOI:** 10.1093/jbmrpl/ziae177

**Published:** 2025-12-06

**Authors:** Gabrielle Stokes, Angela Sheu, Christian M Girgis, Christopher P White

**Affiliations:** Department of Diabetes and Endocrinology, Westmead Hospital, Westmead, NSW, 2145, Australia; Bone and Muscle Research Group, Faculty of Medicine, Nursing and Health Sciences, Monash University, VIC, 3168, Australia; Metabolic Bone Research Group, Centre for Endocrinology and Metabolism, Hudson Institute of Medical Research, Clayton, VIC, 3168, Australia; Bone Epidemiology, Clinical and Translation Science Lab, Skeletal Diseases Program, Garvan Institute of Medical Research, Darlinghurst, NSW, 2010, Australia; School of Clinical Medicine, University of New South Wales, Sydney, NSW, 2052, Australia; Department of Endocrinology, St Vincent’s Hospital, Darlinghurst, NSW, 2010, Australia; Department of Diabetes and Endocrinology, Westmead Hospital, Westmead, NSW, 2145, Australia; Faculty of Medicine and Health, University of Sydney, Sydney, NSW, 2006, Australia; Bone Epidemiology, Clinical and Translation Science Lab, Skeletal Diseases Program, Garvan Institute of Medical Research, Darlinghurst, NSW, 2010, Australia; Department of Endocrinology and Metabolism, Prince of Wales Hospital, Randwick, NSW, 2031, Australia

**Keywords:** hypophosphatemia, iron infusion, hypocalcemia, antiresorptive, fibroblast growth factor 23, calcium-phosphate homeostasis

## Abstract

Intravenous iron infusions (particularly ferric carboxymaltose) are associated with hypophosphatemia. This is mediated by increased fibroblast growth factor 23 (FGF-23), resulting in decreased activation of 25(OH)vitamin D to 1,25(OH)_2_ vitamin D and increased urinary phosphate excretion. Similarly, parenteral antiresorptive agents can lead to hypocalcemia due to reduced bone calcium mobilization, increasing parathyroid hormone (PTH) secretion, and exacerbating kidney phosphate excretion. When given concurrently, electrolyte disturbances can be severe and refractory to treatment, necessitating intravenous replacement, frequent monitoring, and prolonged hospitalization. We describe a case series of six patients with severe hypophosphatemia and hypocalcemia from concurrent administration of intravenous iron and antiresorptive therapy. The average time to hypophosphatemia following iron therapy in the presence of antiresorptives was 17.5 days. This is consistent with the nadir of phosphate 2 weeks following iron infusion and appears to be prolonged and exacerbated by antiresorptive therapy, increasing urinary phosphate loss through increased PTH activity. With the increasing popularity of intravenous iron infusions and parenteral antiresorptive agents, the interplay of these medications is an important consideration for clinicians. The emerging administration of these agents in the community and fragmentation of care across primary and specialist networks create the risk of unintentional concurrent use. Increased awareness of their impact on calcium-phosphate homeostasis is needed to mitigate the risk of severe electrolyte derangements with consideration of alternate iron formulations preferentially in those receiving medications for osteoporosis.

## Background

Severe hypophosphatemia related to parenteral iron preparations is an emerging clinical conundrum, with increasing community use of ferric carboxymaltose (FCM), and to a lesser degree, iron polymaltose.^1–3^ Iron deficiency upregulates hepcidin and hypoxia-inducible factor 1α (HIF1α), regulating, and promoting the transcription of fibroblast growth factor 23 (FGF-23), a hormone derived from osteocytes, regulating phosphate, and vitamin D homeostasis.^4^ Previous studies have shown an increase in FGF-23 cleavage with iron deficiency, driving elevated inactive c-terminal FGF-23, but normal phosphate homeostasis mechanisms are maintained with no change in intact (active) FGF-23 (iFGF-23) levels.^4^ Erythropoietin (EPO) acts to stimulate erythrocyte production and is a key downstream target of HIF regulation.^5^ With iron deficiency, EPO upregulates hepcidin to promote iron store release from erythroblasts and increases circulating FGF-23, both directly and indirectly, of HIF1α.^5^

**Figure 1:**
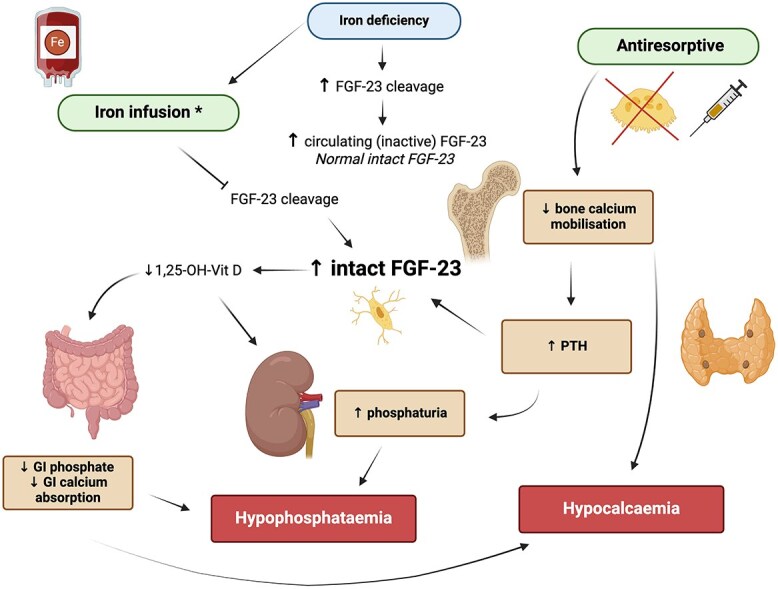
Proposed mechanism of disruption to calcium-phosphate homeostasis by concurrent iron infusion and antiresorptive therapy. Created with Biorender.com. *Most commonly associated with FCM and to a lesser degree, iron polymaltose.

Following intravenous iron administration, the cleavage of FGF-23 is inhibited, increasing circulating active iFGF-23.^1^^,^^4^^,^^5^ As described in Figure 1, increased iFGF-23 decreases the activation of 25(OH)vitamin D to 1,25(OH)_2_ vitamin D. Increased circulating iFGF-23 acts on the kidney to increase urinary phosphate excretion. FGF-23 binds to FGF-receptor 1 in the distal convoluted tubule, in the presence of the α-Klotho co-receptor, to initiate the MAP kinase signaling cascade, leading to the degradation and reduced expression of sodium-phosphate co-transport gene type 2a (NaPi-2a) and type 2c (NaPi-2c), in the proximal renal tubule, promoting phosphaturia.^5^

Additionally, with increased iFGF23, 1,25(OH)_2_ vitamin D is reduced by increased CYP24A1 activity, driving catabolism of active 1,25(OH)_2_ vitamin D to inactive 24,25(OH)_2_ vitamin D_3_. This causes decreased gastrointestinal phosphate absorption, exacerbating hypophosphatemia.^1^ This process has been described as the 6H-syndrome—Hypophosphatemia and Hyperphosphaturia, driven by High FGF-23 with secondary effects of Hypovitaminosis D, Hypocalcemia, and secondary Hyperparathyroidism.^6^

Severe hypocalcaemia following the administration of potent parenteral antiresorptives has also been well-described. In Australia, denosumab, a potent RANK ligand inhibitor, has rapidly increased in popularity since its introduction in 2010 and represents 82% of all osteoporosis therapy reimbursed through the Pharmaceutical Benefits Scheme.^7^ With the use of potent antiresorptive agents, such as denosumab and zoledronic acid, an intravenous bisphosphonate, hypocalcemia can arise due to reduced bone calcium mobilization. This, in turn, increases parathyroid hormone (PTH) secretion, increasing FGF-23 expression and further exacerbating kidney phosphate excretion.^8^ This has predominantly been reported in oncology patients receiving high doses of antiresorptive agents, in patients with pre-existing renal impairment, vitamin D deficiency, or other metabolic bone diseases.^8^^,^^9^

Iron deficiency, with or without associated anemia, and osteoporosis are both common clinical conditions. With the increasing use of potent parenteral antiresorptive and intravenous iron preparations, fragmentation of care across primary and specialist networks creates the risk of unintentional concurrent use. This emerging combination appears to bypass the usual compensatory mechanisms preserving normophosphatemia and normocalcemia, preventing the release of phosphate and calcium in response to intravenous iron load, even in those without underlying metabolic bone disease.^10–16^ When given concurrently, electrolyte disturbances can be severe and refractory to treatment, necessitating intravenous replacement, frequent monitoring, and prolonged hospitalization.

## Case series

We describe a case series of six patients from three hospitals in Sydney, Australia, with severe hypophosphatemia and hypocalcemia in Table 1. All patients were given intravenous iron and antiresorptive therapy within 4 wk of each other. Occurring equally in men and women, the mean age was 74 yr (±17.8 yr).

**Table 1:** Case series of six patients with hypophosphatemia and hypocalcemia due to recent iron and antiresorptive therapy.

	**Age, yr/** **Sex**	**Iron agent**	**Antiresorptive and total duration of therapy**	**Reason for admission**	**Electrolyte nadir and other relevant investigations**	**Electrolytes prior to presentation**	**Management**	**Length of stay, d**
**Patient 1**	68 F	Four doses 100 mg IM iron polymaltose 8 wk prior 1 g IV ferric carboxymaltose 18 d prior	60 mg SC denosumab 22 d prior Three doses of denosumab	Elective orthopaedic surgery	PO4 0.29 mmol/L CCa 2.10 mmol/L FGF-23 65 ng/L FePO_4_ 33.3% “Normal renal function”	PO4 1.0 mmol/L CCa 2.19 mmol/L 25OHD 62 nmol/L PTH 7.7 pmol/L	IV and PO phosphate IV and PO calcium Cholecalciferol	18
**Patient 2**	93 F	1400 mg IV iron polymaltose 27 d prior	60 mg SC denosumab 28 d prior 6 yr of denosumab	Renal hemorrhage + COVID	PO4 *<* 0.10 mmol/L CCa 1.70 mmol/L 25OHD 21 nmol/L PTH 59.5 pmol/L eGFR 72 ml/min/1.73 m^2^	PO4 1.08 mmol/L CCa 2.20 mmol/L	Calcitriol IV and PO phosphate IV and PO calcium Cholecalciferol	17
**Patient 3**	42 F	1000 mg IV ferric carboxymaltose 4 wk prior	5 mg IV zoledronic acid 14 d prior 2 doses of zoledronic acid	Severe symptomatic low phosphate with muscle aches	PO4 0.52 mmol/L CCa 2.18 mmol/L 25OHD 29 nmol/L PTH 17.6 pmol/L TmP/GFR 0.38 mmol/L eGFR 76 ml/min/1.73 m^2^	CCa 2.29 mmol/L Phos 1.42 mmol/L ALP 38 U/L Ferritin 10 μg/L	Calcitriol IV phosphate PO calcium Cholecalciferol	3
**Patient 4**	79 M	1350 mg IV iron polymaltose 21 d prior	60 mg SC denosumab 20 d prior Two doses of denosumab	Colonic stoma bleeding with perioral and peripheral paresthesia	PO4 0.30 mmol/L CCa 1.80 mmol/L 25OHD 41 nmol/L PTH 13.8 pmol/L eGFR >90 ml/min/1.73 m^2^	PO4 1.19 mmol/L CCa 2.29 mmol/L	Calcitriol IV and PO phosphate IV and PO calcium cholecalciferol	3
**Patient 5**	80 M	IV iron infusion 5 d prior^†^	60 mg SC denosumab 2 mo prior 2-3 yr duration of denosumab	Shortness of breath due to anemia from GI bleeding	PO4 0.25 mmol/L CCa 1.98 mmol/L 25OHD 79 nmol/L PTH 17.8 pmol/L 1-25OHD 98 pmol/L eGFR 43 ml/min/1.73 m^2^	N/A	Calcitriol PO phosphate PO calcium Cholecalciferol	7
**Patient 6**	84 M	IV iron infusion 6 d prior^†^	120 mg SC denosumab 6 wk prior	Back pain in setting of metastatic prostate cancer with bone metastases	PO4 0.69 mmol/L CCa 1.84 mmol/L 25OHD 42 nmol/L PTH 33 pmol/L Renal function N/A	N/A	Calcitriol PO phosphate PO calcium Cholecalciferol	18

^†^Details of iron infusion not available.

SC, subcutaneous; IV, intravenous; IM, intramuscular; PO, oral.

PO4, serum phosphate (ref range: 0.75-1.50 mmol/L); 25OHD, serum 25-OH-Vitamin D (ref range: >50 nmol/L).

CCa, serum corrected calcium (ref range: 2.10-2.60 mmol/L); PTH, parathyroid hormone (ref range: 1.6-7.5 pmol/L).

eGFR, estimated glomerular filtration rate; FGF-23, fibroblast growth factor 23 (ref range: 10-54 ng/L).

FePO_4_, fractional excretion of phosphate (ref range: 10%-20%); 1-25OHD, 1-25-OH_2_-Vitamin D (ref range: 60-100 pmol/L).

TmP/GFR, tubular maximum reabsorption of phosphate to glomerular filtration rate (ref range: 0.84-1.23 mmol/L).

Due to the severity of electrolyte disturbance, all patients required admission to hospital or prolongation of hospital admission. The mean length of hospitalization was 11 d (± 7.46 d).

The average time to hypophosphatemia following iron administration was 17.5 d (5-28 d). Antiresorptive therapy was given 31 d prior, on average (14-60 d). The interval between intravenous iron and antiresorptive therapy varied between 1 d and 7 wk, indicating a prolonged risk of interaction. Previous reports of the interaction between intravenous iron therapy and parenteral antiresorptive have focused on FCM, which has higher rates of hypophosphatemia compared to other widely available iron preparations, and denosumab, with reported hypocalcemia rates of up to 6.3% of patients in a real-world setting.^17^ Our case series is the first to highlight that this interaction can occur with varied forms of parenteral antiresorptives, including zoledronic acid and iron formulations.

Urinary phosphate wasting was confirmed in two patients. Patient 1 had severely low serum phosphate at 0.29 mmol/L with a corrected calcium of 2.10 mmol/L. Her FGF-23 levels were elevated at 65 ng/L (Normal range: 10-54 ng/L), and fasting urine fractional excretion of phosphate (FePO_4_) was increased to 33.3% (Normal range: 10%-20%). Following treatment with intravenous and oral electrolyte replacement, her phosphate, FePO_4_, and iFGF-23 levels normalized.

Patient 3 had a history of renal tubular acidosis and following concomitant administration of intravenous iron and zoledronic acid, presented with symptomatic hypophosphatemia. Despite maximal oral replacement in a community setting, serum phosphate was 0.52 mmol/L, corrected calcium was 2.18 mmol/L, and tubular maximum reabsorption of phosphate to glomerular filtration rate (TmP/GFR) was decreased to 0.38 mmol/L (0.84-1.23), consistent with renal phosphate wasting.

## Discussion

We describe multiple cases of severe hypophosphatemia and/or hypocalcemia following the administration of parenteral iron and antiresorptive therapies in close proximity. The average time to hypophosphatemia following iron and antiresorptive therapy was 17.5 d (6-28 d). This is consistent with the nadir of phosphate 2 wk following iron infusion and appears to be prolonged and exacerbated by antiresorptive therapy, increasing urinary phosphate loss through increased PTH activity.^16^^,^^18^

Severe hypocalcemia has been well described in patients receiving antiresorptives—particularly denosumab.^1^^,^^16^ This has predominantly been reported in oncology patients receiving high doses and patients with pre-existing renal impairment.^9^^,^^18^ However, the pathogenesis driving hypocalcemia with antiresorptive therapy is not unique to these populations, and our patient cohort largely received treatment for osteoporosis with no other risk factors for refractory electrolyte disturbances. When antiresorptives are co-administered with intravenous iron, there appears to be a loss of the usual homeostatic mechanisms, preventing the release of phosphate and calcium in response to intravenous iron load, even in those without underlying metabolic bone disease.

The mechanism of hypophosphatemia following parenteral iron administration is well-described via a disruption of FGF-23-mediated phosphate homeostasis, resulting in increased urinary phosphate excretion.^1^ However, this phenomenon is variably documented in clinical trials and in scientific literature, with the rates of hypophosphatemia ranging from 0%-92% due to inconsistent reporting of adverse effects.^19^ FCM is an intravenous iron preparation with a non-dextran stable carbohydrate shell, decreasing the risk for dextran-associated hypersensitivity reactions.^20^ The stability of this carbohydrate shell is postulated to drive a slower release of iron and lower levels of labile (unbound) iron that can cause cell toxicity.^20^ This preparation, in particular, however, is associated with an increased risk of hypophosphatemia, especially in the setting of normal renal function.^19^

Alerts have been issued through the US Food and Drug Administration, European Medicines Agency, and Australian Therapeutic Goods Administration agencies alerting clinicians to the risk of significant hypophosphatemia with FCM and the potential for associated osteomalacia, especially with repeated doses.^21–23^ However, the interaction of FCM and potent antiresorptive agents has not yet been identified as a risk despite the potential for significant clinical interaction.

In contrast, ferric derisomaltose (FDI) has been shown to be less likely to induce severe hypophosphatemia with rates of 5% in one pooled meta-analysis, compared to 51% in those treated with FCM.^19^ Though less widely available, FDI may be preferred in those with previous severe FCM-induced hypophosphatemia or with significant risk factors.^1^^,^^24^^,^^25^ Iron sucrose may have decreased risk of hypophosphatemia, though reported rates vary between 0%-40%.^26–29^ Ferumoxytol and low-molecular-weight iron dextran appear to have lower rates of hypophosphatemia (0.9% and 0%, respectively) but have been associated with higher risk of anaphylaxis compared to iron sucrose or FCM.^4^^,^^30^^,^^31^ The differing rates, severity, and duration of hypophosphatemia between IV iron formulations is postulated to be related to the specific pharmacological properties of the iron-carbohydrate complex but this requires further research to fully elucidate.^19^ The specific composition of the carbohydrate moiety, separate from iron, is difficult to study and isolate due to the heterogeneity of manufacturing processes.^19^

The co-prescription of parenteral iron and osteoporosis therapies presents a management dilemma. With increasing fragmentation of care across primary and specialist networks, the risk of unintentional concurrent administration is increased, and clinicians should be aware of the need to proactively ask about a history of iron therapy or antiresorptive use when considering prescription.

With an increasing understanding of the risks of intravenous iron therapy, clinicians should consider the need for parenteral administration carefully, particularly in patients on antiresorptive therapy. Where safe and feasible, oral iron therapy may be preferred, and if parenteral iron is required, lower-risk formulations, such as FDI, should be considered due to their lower association with severe hypophosphatemia.^1^^,^^19^^,^^24^^,^^25^

Clinicians should be alert to the need for monitoring of calcium and phosphate when antiresorptive agents and parenteral iron are given within 4 wk of each other. There have been multiple case reports of the interaction between intravenous iron infusions—predominantly FCM—and denosumab, an antiresorptive agent.^10–16^^,^^32^ Many of these case reports originate from Australia due to the high prescribing rates of both agents. More investigation into the frequency of the therapeutic interaction and optimal time between iron and antiresorptive therapy is needed.

As the nadir of phosphate is usually seen 2 wk following iron infusion and similarly, the nadir in calcium seen 2 wk following denosumab use, we suggest monitoring serum calcium and phosphate at 7, 14, and 28 d when co-administration of these agents has occurred. Other factors that may exacerbate risk of hypophosphatemia and hypocalcemia should be considered when monitoring electrolytes with measurement and supplementation of 25-OH-Vitamin D and magnesium (due to impaired PTH secretion in the presence of hypomagnesemia) if deficient.

In patients with significant risk factors for hypophosphatemia and hypocalcemia, such as chronic kidney disease, pre-infusion monitoring of phosphate, calcium, magnesium, renal function, and 25-OH-Vitamin D should be undertaken. Prophylactic replacement of calcium, phosphate, and/or calcitriol can be considered under expert supervision. Calcitriol should be used with care, as it increases urinary calcium excretion and can increase the risk of renal stone formation. This risk may be further exacerbated with iron-induced hypophosphatemia by the presence of hyperphosphaturia and in those with pre-existing risk factors such as inflammatory bowel disease driving hyperoxaluria.^33^

Whilst electrolyte disturbance has occurred within 4 wk in many of these case reports, including some of our cases, patients 5 and 6 had significant hypophosphatemia and hypocalcemia up to 8 wk following antiresorptive therapy (in combination with more recent intravenous iron). This raises the concern that the period of monitoring may need to be extended further, perhaps to 8 wk, particularly in the presence of identified risk factors, such as high-dose denosumab.

In patients on long-term denosumab, delay or discontinuation risks rebound-associated fractures, and in this cohort, high-risk iron formulations such as FCM should be avoided, where possible, and substituted with FDI.^34^ Zoledronic acid infusion should be deferred, where possible, given its long half-life and incorporation into the bone matrix. Burosumab, a human recombinant monoclonal antibody that binds FGF23, has been successfully used in refractory iron-induced hypophosphatemic osteomalacia in one case report.^35^

Further research is required to determine the period of highest risk and optimal management of the co-administration of these agents, given difficulties in deferring treatment.

## Conclusion

Parenteral iron infusions, particularly FCM and potent antiresorptive therapies, both have the potential to interrupt calcium-phosphate homeostasis, driving prolonged and severe electrolyte disturbance. The emerging administration of these agents in the community and fragmentation of care across primary and specialist networks creates the risk of unintentional administration in close proximity, with associated risks of severe and prolonged hypocalcemia and hypophosphatemia. Increased awareness of the impact of the concurrent use of parenteral iron and antiresorptive agents on calcium-phosphate homeostasis is needed to mitigate the risk of severe electrolyte derangements.

## Data Availability

Due to its potentially identifying nature, supporting data is not available to be shared publicly.
